# Outcome measurement in mental health services: insights from symptom networks

**DOI:** 10.1186/s12888-019-2175-7

**Published:** 2019-06-28

**Authors:** Guillaume Barbalat, Don van den Bergh, Jolanda Jacqueline Kossakowski

**Affiliations:** 1 Counties Manukau District Health Board, Auckland, New Zealand; 2 Department of Psychology, University of Amsterdam, Amsterdam, the Netherlands

**Keywords:** Outcome measurement, Community mental health services, Individual scores, Symptom networks, HoNOS

## Abstract

**Background:**

In mental health, outcomes are currently measured by changes of individual scores. However, such an analysis on individual scores does not take into account the interaction between symptoms, which could yield crucial information while investigating outcomes. Network analysis techniques can be used to routinely study these systems of interacting symptoms. The present study aimed at comparing outcomes using individual scores vs. symptom networks, after a 1 year intervention at a local community mental health centre.

**Methods:**

We used the Health of the Nation Outcomes Scales, which defines a set of 12 scales investigating mental health and social functioning. We first assessed how individual scores varied from baseline to end point and which items were associated to treatment response. Second, using network analysis techniques, we measured the overall connectivity of the networks and determined the most important symptoms.

**Results:**

The individual scores analysis revealed a significant improvement amongst most scales. No specific factors were related to treatment response at end point. At end point, network analysis revealed a very densely connected network while agitation and substance use were the most connected symptoms.

**Conclusions:**

Individual scores and symptom network analysis resulted in very different outcomes, with network analysis toning down positive results gained from individual scores analysis. The strong connectivity of patients’ network at end point may reflect their increased complexity. Allocating more resources to interventions tailored to symptoms that are the most connected would decrease network connectivity and improve patients’ prognosis. When investigating outcomes, network analysis could give insights complementary to standard analysis on individual scores.

**Electronic supplementary material:**

The online version of this article (10.1186/s12888-019-2175-7) contains supplementary material, which is available to authorized users.

## Background

It is crucial in mental health to routinely obtain clinical outcome measures against which the quality and effectiveness of services can be monitored, judged and improved [1]. Currently, outcomes are related to individual scores (or individual sum scores) that quantify the severity of patients’ mental health symptoms or disorders [2]. To judge the effectiveness of a treatment program, services are interested in assessing how those scores vary from baseline to the end of interventions and what variables are associated with recovery vs. treatment failure [3]. Subsequently, organizations could re-define or put more resources on interventions designed to improve factors significantly associated to treatment failure.

However, investigating outcomes based on individual scores or sum scores does not take into account the fact that symptoms of mental disorders reliably influence each other [4–6]. For instance, in the case of a major depressive disorder, a patient might first develop difficulties sleeping, after which she experiences tiredness and cognitive impairment, which results in feelings of worthlessness, sadness and lack of interest. Simply adding up those symptoms to an episode of depression does not take into account the causal chain that led to this episode, which could be of particular importance when investigating and treating patients’ disorders.

Such causal relations between symptoms can be modelled using the framework of symptom networks [7–9]. The network perspective posits that an “active” causal chain of symptoms, where one symptom leads to another, reflects the progression of a disorder: the more connected the network, the more active the disorder [10]. An important treatment goal could then be to decrease the overall connectivity of patients’ network of symptoms with treatment interventions. In addition, considering disorders as networks also allows the assessment of the importance of particular symptoms for a given network, that is, the degree to which symptoms are connected to others [11]. The idea then becomes to address those key symptoms in priority in order to bring the whole disorder down – like a house of cards –, rather than tackling symptoms in a blind manner. Such an analysis would be a key asset in the perspective of measuring outcomes, as this could help redefine and tailor therapeutic interventions to those symptoms that are core to the networks.

Using network analysis techniques, such systems of interacting symptoms can be routinely represented, analysed, and studied in their full complexity [12, 13]. First, network estimation allows an optimal graphical representation of the relationships between symptoms in the network, i.e. it gives an account of the overall network connectivity. Second, centrality analysis measures the degree to which a particular symptom is involved in the network in terms of its connectivity to the other symptoms.

The present study aimed at comparing outcomes based on individual scores analysis vs. symptom networks analysis, after a one-year intervention at a local community mental health centre (CMHC). We first assessed how individual scores varied from baseline to end point and which items were associated to treatment response or treatment failure. Second, we demonstrated the additional value of network analysis when investigating service outcomes by measuring the overall connectivity of the networks (network estimation) and determining which were the most important symptoms (centrality analysis). We used the Health of the Nation Outcomes Scales (HoNOS; [14]), which is widely used across the world and defines a set of 12 scales investigating health and social functioning of people using mental health services.

## Methods

### Study sample (Fig. 1)

The setting for the present study was a local CMHC situated in the eastern region of Auckland, New Zealand. The service serves a population of approximately 147,000 local residents. Cases were identified retrospectively by performing computer searches of our current caseload of 433 service users.

**Fig. 1:**
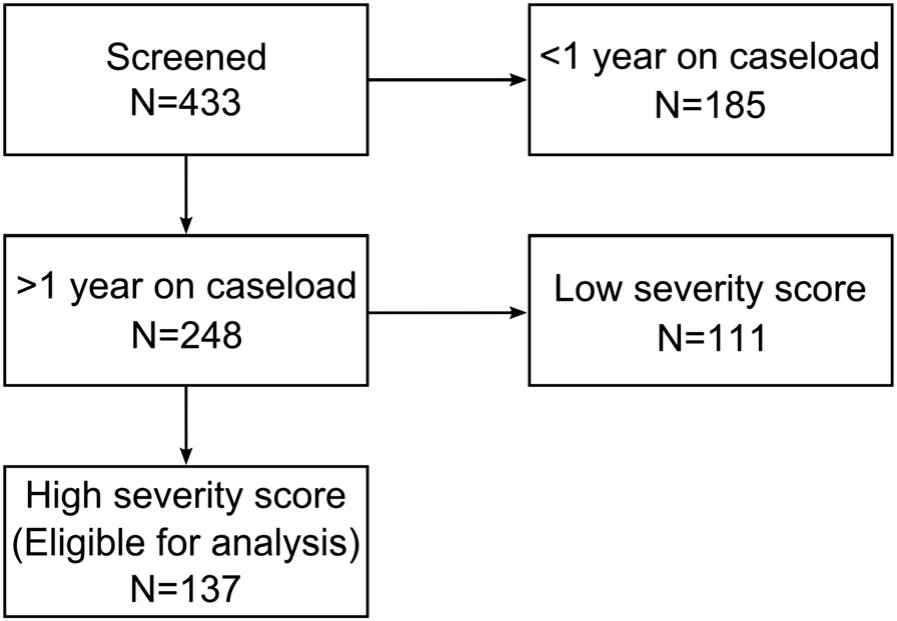
Study sample flow chart

The intended timeframe was 1 year from entry to the service, irrespective of the date of entry. This timeframe usually leaves enough time for our interventions to affect patients’ issues with consistency. Initial database screening identified 248 patients who satisfied this criterion.

Diagnosis was not a criterion for inclusion or exclusion: any patient receiving secondary care for mental health disorders during the study time frame was eligible for inclusion. However, we did exclude patients who did not reach criteria for severity at baseline from our analysis (see below for a definition of this severity criteria based on HoNOS scores). Indeed, for these patients, our interventions may have only had little or marginal effects and including them in our analysis would have not reflected the real impact of our interventions on service outcomes. For instance, some patients had their treatment already started under a different team (e.g. our local crisis team) and their mental health had already started to improve significantly before being treated by our team. From the 248 patients mentioned above, a total number of 111 patients did not reach severity at baseline and thus were removed from the analysis. Hence, the final sample comprised a total of 137 cases.

Patients’ socio-demographic characteristics are described in Table 1. The medical ethics boards of the participating centre approved the study.

**Table 1:** Socio-demographic characteristics

Age; range; mean (SD)	18–70; 41.1 (13.7)	
Gender; male (%)	56 (40.9%)	
Ethnicity; N (%)	European	90 (65.7%)
	Asian	26 (19.0%)
	Maori	7 (5.1%)
	Pacific Island	6 (4.4%)
	African	5 (3.6%)
	Other	3* (2.2%)
DSM-IV diagnosis; N (%)	Mood disorder	54 (39.4%)
	Psychotic disorder	41 (29.9%)
	Anxiety disorder	22 (16.1%)
	Borderline personality disorder	10 (7.3%)
	Autism spectrum disorder	6 (4.4%)
	Other	4** (2.9%)

* Other ethnicity: one patient was of a hispanic ethnicity and 2 did not have their ethnicity recorded

**Other primary diagnosis: one patient was diagnosed with ADHD, one with mental retardation, one with substance use disorder and one did not have her primary diagnosis recorded

### Our CMHC interventions

The interventions are tailored to the patients’ needs and are in line with the so-called bio-psycho-social model of clinical care. They involve psychiatrists, clinical psychologists, occupational therapists, nurses, alcohol and drug clinicians, social workers, supported employment consultant, peer support specialists, and cultural support staff.

### HoNOS scores

The HoNOS is a clinician rated tool used to measure the health and social functioning of people using mental health services [14]. The HoNOS was published by the Royal College of Psychiatrists in 1996 and is now the most widely used outcome measure in specialist mental health services in England and overseas. It was developed as a means of recording progress towards the UK “Health of the Nation” target to improve significantly the health and social functioning of mentally ill people. The scales were developed using stringent testing for acceptability, usability, sensitivity, reliability and validity.

The HoNOS is a set of 12 scales, each measuring a type of problem commonly presented by patients in mental health care settings. A completed HoNOS score sheet provides a profile of severity rating of 12 different scores. The scales cover a wide range of health and social domains - psychiatric symptoms, physical health, functioning, relationships and housing:

1: Overactive, aggressive, disruptive or agitated behaviour.

2: Non-accidental self-injury.

3: Problem drinking or drug-taking.

4: Cognitive problems.

5: Physical illness or disability problems.

6: Problems associated with hallucinations and delusions.

7: Problems with depressed mood.

8: Other mental and behavioural problems. Note that 80% of our sample had this item related to anxiety.

9: Problems with relationships.

10: Problems with activities of daily living.

11: Problems with living conditions.

12: Problems with occupation and activities.

Severity is measured on a five-point severity scale (0,1,2,3,4):

0: no problem within the period rated; 1: sub-threshold problem; 2: mild but definitely present; 3: moderately severe; 4: severe to very severe.

Brief examples of each rating point are given for each of the 12 scales in the glossary which is used alongside the score sheet [15] (see also Additional file 1). The rating period is generally 2 weeks preceding the assessment for all clients of community based services. The scales are completed after routine clinical assessments on the basis of all information available to the rater (whatever the source). Raters were psychiatrists or other mental health clinicians who had 1 day training. Once staff is trained, the actual 12 ratings take, on average, about 4 min.

Determining disorder severity based on HoNOS scores has been a matter of debate. Adding up the scores of all 12 scales may not be particularly informative as they are wide in their coverage. Instead, others have argued for an index of severity based on individual scores ([16] modified by [17]; see also [18]). Following these recommendations, we defined patients’ disorders as not severe if no items were rated 3 or above. In contrast, patients’ disorders were defined as severe if at least one item was rated 3 or above. All recruited patients reached criteria for severe disorders (see above). We defined our criteria for treatment response as reaching the non-severity level. Conversely, non-response was defined as still presenting a severe level of disorder.

### Statistical analysis

Our analysis included 2 sets of (individual) HoNOS scores: (1) at baseline, i.e. when patients were first seen by our team; (2) 1 year after entry to the service (which we defined as the “end point”). However, HoNOS at end point could not always be strictly completed 1 year after referral to our service (e.g. because some patients were temporarily difficult to locate; mental health clinicians usually completing HoNOS were on leave etc.). We chose to select the HoNOS that was completed as close as possible to the 1 year mark. Overall, the delay between measurements at baseline and end point was of 462.1 days (SD 170.3 days).

#### Symptom to symptom differences

A Wilcoxon rank sum test for paired ordinal data was performed to test differences for each scale at baseline vs. end point. We also calculated the proportion of patients reaching treatment response (patients reaching the non-severity level: no HoNOS items rated 3 or above) at end point. We used a logistic regression model to evaluate the potential relation between reaching treatment response with HoNOS items at baseline, age and gender.

#### Network estimation

A network conceptualizes HoNOS as a system of mutually interacting HoNOS items [6]. Such networks contain nodes (individual HoNOS items) and edges (associations among individual HoNOS items [19]). Here, network structures of HoNOS scores at baseline and end point were estimated separately.

One very simple possibility to represent edges would be to use correlation coefficients between nodes. However, this may often reflect spurious relationships that disappear when the other nodes in the network are conditioned on. For this reason, we modelled the conditional dependencies between HoNOS items in which an edge indicates a nonzero partial correlation between two nodes, while controlling for all other nodes in the network [20].

As is often the case in network analysis, our network model contained a very high number of potential edges (2^12^ edges). We controlled for false positive edges due to multiple testing by using the least absolute shrinkage and selection operator (lasso; [21]). This procedure guarantees shrinkage of partial correlations such that very small edges (likely due to noise) are pushed to zero and thus removed from the network. This encourages the selection of simple, sparse models (i.e. with fewer edges). The amount of shrinkage is defined by a tuning parameter lambda used in the lasso procedure.

The optimal sparse network model that fits the data using lasso is obtained by minimizing the extended Bayesian information criterion (EBIC; [22]). The EBIC penalizes maximum likelihood estimation by taking into account both the number of edges and the complexity (size) of the model space. The strength of the latter penalty depends on the value of a so-called hyperparameter gamma which, in the current study, we chose to set to zero (as a higher value would have resulted in very few edges or none at all). Note that this means that we selected the optimal network model with the ordinary BIC instead of the extended BIC.

We decided to regress out the influence of the time period between baseline and end point on HoNOS scores and use the residuals for subsequent network estimation. The rationale for this procedure is twofold. First, as mentioned above, even though we were careful to collect HoNOS scores that were measured around 1 year after the initial HoNOS measurement, we noted variation in the period between both ratings. Second, we anticipated that HoNOS scores would improve from baseline to end point. This in turn would have narrowed the range of HoNOS scores and artificially changed the correlations between symptoms. We reasoned that by regressing out the time period between baseline and end point, the variability of HoNOS scores at end point would be comparable to that at baseline.

We used the nonparanormal SKEPTIC transformation to estimate the correlation matrix of the residuals at baseline and end point [23]. This method exploits nonparametric rank-based correlation coefficient estimators (including Spearman’s rho and Kendall’s tau) and is used to relax the normality assumption of the Gaussian Graphical Model.

#### Difference in overall connectivity

Overall connectivity can be summarized by global strength and is defined as the weighted absolute sum of all edges in the network [24]. We checked for differences in overall connectivity by means of the network comparison test (NCT) developed by van Borkulo et al. [25]. This 2-tailed permutation test randomly regroups individuals from the baseline sample and the end point sample repeatedly (1000 times) and calculates the differences in global strength between those samples [26]. The resulting distribution under the null hypothesis (both samples are equal) is used to test the observed difference of the original samples against a significance level of 0.05.

#### Centrality analysis

To gain more insight on the importance of individual HoNOS items in the networks, we used the measure of node strength [27]. Node strength is a centrality measure that calculates the weighted number of connections of a focal node and thereby the degree to which that node is involved in the network.

We determined the stability of node strength at baseline and end point by means of bootstrapping procedures [28]. We chose to only interpret measures that satisfied the stability criteria. Note that node strength was shown to be the more stable of a set of 3 centrality measures also including closeness and betweenness (for a definition of those centrality measures, please refer to [27]) in a recent study [28].

Statistical analysis was performed using R, version 3.3.1 (R core Team, 2017).

## Results

### Symptom to symptom differences

Each HoNOS score was significantly lower at end point vs. baseline (*p* < 0.02) except “Physical illness or disability problems” (*p* = 0.13) and “Problems with living conditions” (*p* = 0.80; see also Table 2). Overall, 82 patients of the original sample (60%) reached criteria for treatment response (i.e. no items were rated 3 or above) at end point, and 55 patients (40%) did not reach this criteria (i.e. at least one HoNOS item was rated 3 or above). A logistic regression model did not identify age, gender, or any HoNOS items at baseline as predictors of reaching treatment response at end point.

**Table 2:** Analysis of individual HoNOS scores at baseline and end point

HoNOS item	Baseline; mean (SD)	End point; mean (SD)	Statistics (Wilcoxon, *p* value)
1 Overactive, aggressive, disruptive or agitated behaviour	0.87 (1.1)	0.32 (0.53)	V = 2290, *p* < 0.001
2 Non accidental self-injury	0.69 (1.05)	0.33 (0.68)	V = 1202, p < 0.001
3 Problem drinking or drug-taking	0.53 (0.99)	0.20 (0.55)	V = 755.5, p < 0.001
4 Cognitive problems	0.61 (0.85)	0.30 (0.53)	V = 1696.5, p < 0.001
5 Physical illness or disability problems	0.76 (1.13)	0.80 (1.06)	V = 1133.5, p = 0.80
6 Problems associated with hallucinations and delusions	1.01 (1.40)	0.48 (0.83)	V = 1500.5, p < 0.001
7 Problems with depressed mood	1.68 (1.12)	1.01 (1.06)	V = 3753.5, p < 0.001
8 Other mental and behavioural problems	1.96 (1.31)	1.61 (1.17)	V = 3088, *p* = 0.008
9 Problems with relationships	1.58 (1.08)	1.01 (0.99)	V = 3124, p < 0.001
10 Problems with activities of daily living	0.99 (1.01)	0.77 (0.93)	V = 1572, *p* = 0.02
11 Problems with living conditions	0.33 (0.69)	0.23 (0.56)	V = 647, p = 0.13
12 Problems with occupation and activities	0.92 (1.16)	0.51 (0.95)	V = 1821.5, p < 0.001

### Network analysis

For this analysis, we used the residuals of a regression of the time period between baseline and end point on HoNOS scores rather than actual HoNOS scores. As anticipated, none of the paired t tests comparing residuals at baseline vs. end point were significant (t < 0.06, *p* > 0.95).

**Fig. 2:**
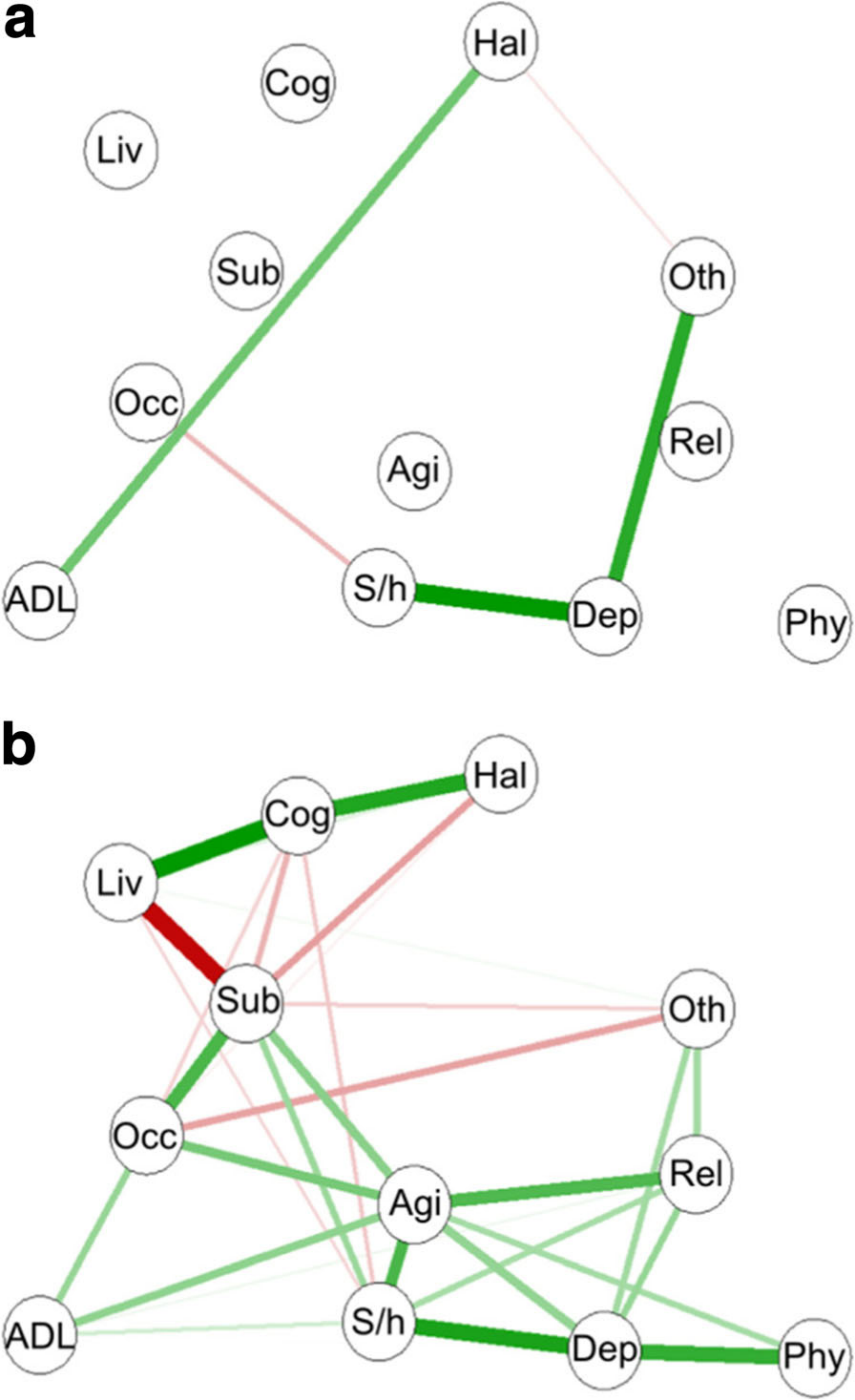
Network structures of HoNOS items at baseline (**a**) and end point (**b**). Green lines represent positive partial correlations, whereas red lines represent negative partial correlations. Thicker edges represent stronger associations (positive or negative). Agi: Overactive, aggressive, disruptive or agitated behaviour; S/h: Non accidental self-injury; Sub: Problem drinking or drug-taking; Cog: Cognitive problems; Phy: Physical illness or disability problems; Hal: Problems associated with hallucinations and delusions; Dep: Problems with depressed mood; Oth: Other mental and behavioural problems; Rel: Problems with relationships; ADL: Problems with activities of daily living; Liv: Problems with living conditions; Occ: Problems with occupation and activities

Figure 2a,b shows network of HoNOS items at baseline and end point, respectively. Edges between nodes within a network correspond to partial correlations between items, controlling for all other items. Each node corresponds to a single HoNOS item (as given in Table 2). The stronger a connection between two nodes, the thicker the edge. Positive and negative connections are denoted by green and red edges, respectively. The graphical representation of networks is based on the Fruchterman-Reingold algorithm that places nodes with stronger and/or more connections closer together [29]. Note that, for representation purposes, we used the same layout for networks at end point (Fig. 2b) and at baseline (Fig. 2a).

As shown in Fig. 2a, the network at baseline was scarce. Main connections were between “non-accidental self-injury” and “problems with depressed mood”; “problems with depressed mood” and “other mental and behavioural problems”; “hallucination and delusions” and “problems with activities of daily living”.

In contrast, Fig. 2b revealed a much more connected network at end point. Significant connections were observed within mental health symptoms (e.g. “Overactive, aggressive, disruptive or agitated behaviour” and “Problem drinking or drug-taking”; “Overactive, aggressive, disruptive or agitated behaviour” and “Non accidental self-injury”), between mental and social symptoms (e.g. “Overactive, aggressive, disruptive or agitated behaviour” and “Problems with relationships”; “Problem drinking or drug-taking” and “Problems with occupation and activities”), and within social symptoms (“Problems with activities of daily living” and “Problems with occupation and activities”).

### Differences in overall connectivity between networks

We then compared the overall connectivity between networks (at baseline vs. end point). Our analysis using the NCT revealed that both networks are statistically dissimilar in overall connectivity (*p* = 0.03), confirming the visual impression that the symptom network at end point was more strongly connected than that at baseline (Fig. 2).

### Centrality analysis

**Fig. 3:**
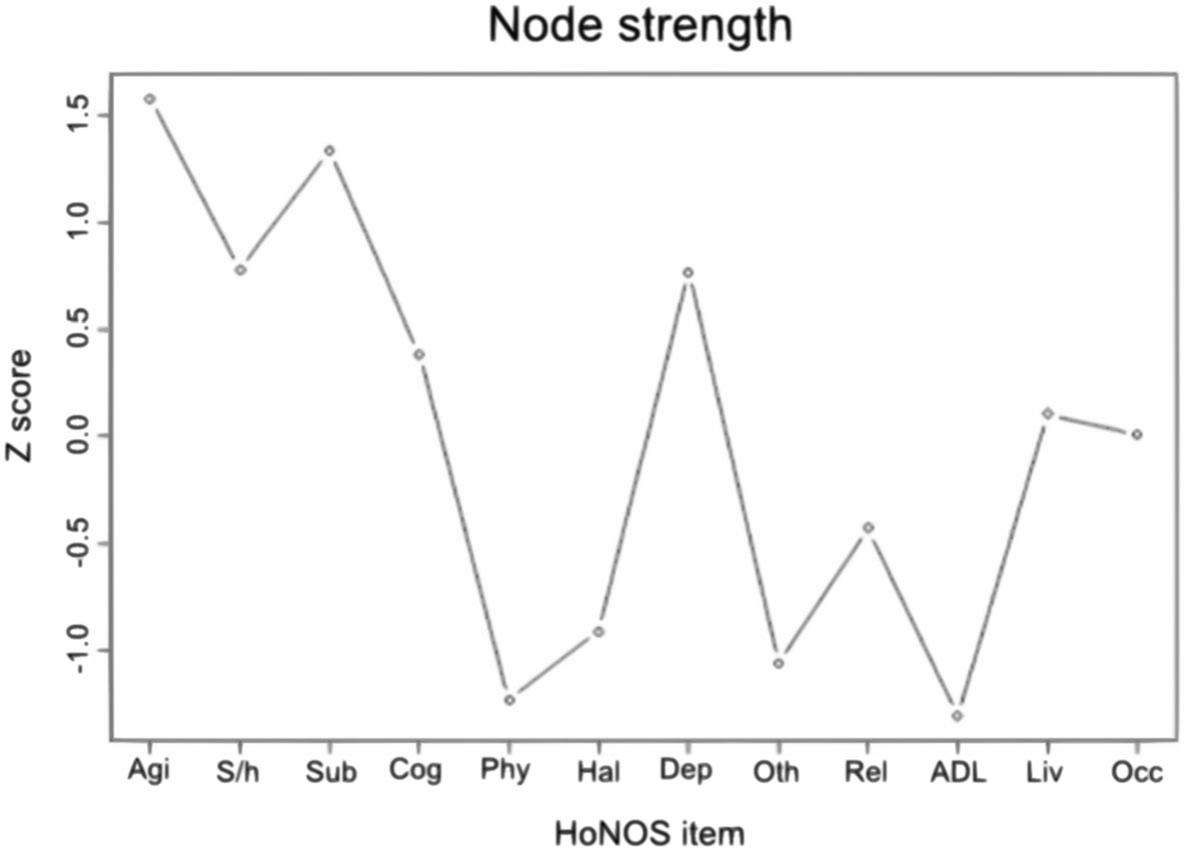
Node strength for network at end point. z scores of standardized node strength of each HoNOS item for network at end point. See Fig. 2 legend for definitions of abbreviated terms

Node strength satisfied the stability criteria at end point but not at baseline. The 2 most central nodes at end point were “Overactive, aggressive, disruptive or agitated behaviour” and “Problem drinking or drug-taking” (Fig. 3). The 2 least central nodes were “Physical illness and disability problems” and “Problems with activities of daily living” (Fig. 3). Note that none of the other centrality measures (closeness and betweenness) satisfied the stability criteria at baseline or at end point.

## Conclusions

The aim of the current study was to assess treatment outcomes from a cohort of patients with severe mental health issues followed up for a period of 1 year in a local CMHC. We used the HoNOS, a widely used clinician rated scale which evaluates 8 cardinal psychiatric symptoms as well as 4 social items on a five-point severity scale [14]. We used 2 different methods to investigate outcomes: (1), standard individual scores analysis (i.e. how each HoNOS score changed from baseline to end point); and (2), symptom network analysis [9], where patients’ issues are conceptualized as networks of connected symptoms (i.e. how the *association* of HoNOS scores changed from baseline to end point).

Our individual scores analysis revealed a significant improvement amongst most scales (only items related to physical issues as well as living conditions did not improve – items that our interventions do not directly target). This result is somewhat balanced out by the fact that 40% of patients did not respond to our interventions. Unfortunately, a logistic regression did not identify any obvious factors that significantly differentiated between patients who responded vs. those who did not.

From our symptom network analysis, we found that HoNOS items were more connected at end point than at baseline. While this finding needs replication and cautious interpretation, it suggests that network analysis can bring out very different insights than traditional analysis on individual scores when investigating outcomes. In the following, we will discuss those insights, including potential implications and ideas for future research.

At baseline, decreased connectivity of symptoms could stem from the clinical heterogeneity of our patients group [30, 31]. Indeed, at entry to the service, the fact that patients presented with various clinical manifestations would result in decreased interrelations between HoNOS transdiagnostic items. Conversely, clinical improvement at end point would reduce the variability between HoNOS items and, as compared to baseline, increase their interrelations. That diagnoses heterogeneity decreases network connectivity when using transdiagnostic scales such as HoNOS, and that clinical improvement then increases connectivity would be interesting hypotheses to test in future studies. However clinical homogeneity alone is unlikely to give an account for the strong connectivity of our patients’ network at end point. Indeed, despite testing homogeneous samples, previous studies have shown various grades of connectivity amongst symptom networks [32–37].

Another interesting hypothesis could explain patients’ high degree of connectivity at end point. Indeed, seen from the perspective of symptom networks, this result suggests that our patients suffer from rather advanced disorders. The fact that our patients’ networks are active 1 year after treatment has started reveals that causal relations between symptoms are sufficiently strong and self-sustaining to resist standard psychiatric treatment. This hypothesis seems to tone down the results from our individual scores analysis, where individual symptoms improved from baseline to end point and a significant proportion of patients reached the non-severity level at end point. As such, this may reveal that a wider view of patients’ disorders as reflecting dynamic interplays between symptoms – and not only a sum of individual symptoms that do not influence each other – is warranted when investigating outcomes and clinical interventions efficacy. Future studies can leverage the opportunity to further understand network changes over time, specific treatments that can de-activate them and their effects on patients’ clinical states.

Such a strong connectivity of our patients’ network at end point may reflect the complexity of their disorders and their high risk of relapse. Indeed, a previous report investigating symptom network structure in association with the course of depression showed that patients with persistent symptoms had a more connected network than those who remitted [10]. It is noteworthy however that this was not confirmed in a later report where the difference in connectivity between those with persistent vs. remitted symptoms failed to reach statistical significance [38]. A potential goal for future studies could be to further investigate such network changes (and their potential causes) for responders vs. non responders, both at baseline and at end point. Unfortunately, we were unable to perform such an analysis in the current study because our samples of responders vs. non-responders were too small.

A potential goal in terms of treatment priorities would then be to allocate more resources to interventions specifically designed to decrease the overall connectivity of the patients’ network. One way to achieve this goal is by tailoring those interventions to symptoms that are most central to the network. This would lead to cascading effects on other symptoms and reduce their correlations. In turn, this would decrease the risks of relapse and improve outcomes in a cost effective way. In the current study, agitation and problems with drug and alcohol were the 2 most important symptoms at end point. Based on those findings, more resources could be allocated on programs to decrease agitation (e.g. by training staff to use relaxation methods, sensory modulation and dealing with distress skills) and substance use (e.g. by training staff to use motivational interviewing and brief interventions).

## Limitations

First, our sample was heterogeneous in terms of patients’ diagnoses and intervention types. We cannot rule out the fact that outcomes would have been different if patients were more homogeneous in terms of their diagnoses or the interventions they received. However, our study aimed at comparing service level outcomes using two different methods of analysis, irrespective of diagnoses or interventions. Because the sample at baseline was strictly identical to that at end point, there is no reason to believe that variations in diagnoses and interventions could have influenced outcomes differently using one method of analysis vs. the other. Then, it is very unlikely that our main finding, namely the fact that the symptom network analysis gave different insights than the individual scores analysis, could be explained by diagnosis heterogeneity or intervention type. Besides, note that the symptom network model itself challenges the concept of mental health diagnoses in that it aims at explaining mental health issues by the inter-relations between symptoms rather than some form of underlying entity [4, 39].

Second, in the absence of a control group, we cannot rule out that some of our results could simply be a reflection of the inherent variability of patients’ symptoms scores over time. For instance, patients may have entered the service at a time when their symptoms were unstable and would naturally tend to be closer to their average when measured at end point. However, such a regression to the mean could again not explain why outcomes based on symptom network analysis led to different insights than those based on individual scores analysis.

A third limitation of the current study was that, with only 12 items, the HoNOS only gives partial insights on patients’ psychopathology. However, the brevity of HoNOS completion and the fact that it is thought to reflect the core of mental health issues make the HoNOS a satisfactory and suitable tool to explore outcomes in mental health [40].

Despite these potential limitations, we showed that symptom network analysis could give interesting insights on services’ specific needs and objectives, complementary to the traditional pre-post analysis of symptom to symptom changes. Investigating mental health services outcomes using an individual scores approach may be limited by the fact that psychiatric disorders are probably best conceptualized as dynamic interplays between symptoms. Such a conceptualization calls for new ways to evaluate outcomes in mental health where potential goals could be to reduce network connectivity and tackle symptoms that are more connected to others. To our knowledge, this is the first study to describe how symptom networks could be used to gather information about treatment outcomes at the service level and re-define treatment priorities.

## Additional file

Additional file 1: Glossary for HoNOS Score Sheet. (PDF 353 kb)

## Data Availability

Research data can be accessed on request to the corresponding author (guillaumebarbalat@gmail.com).

## Abbreviations

BIC: Bayesian Information Criterion.
CMHC: Community Mental Health Centre.
EBIC: Extended Bayesian Information Criterion.
HoNOS: Health of the Nation Outcomes Scales.
LASSO: Least Absolute Shrinkage and Selection Operator.
NCT: Network Comparison Test.
SKEPTIC: Spearman/Kendall Estimates Preempt Transformations to Infer Correlation.
