# Supplementary material for: Outcome measurement in mental health services: insights from symptom networks
Source: BMC Psychiatry. 2019 Jun 28;19:202. doi: 10.1186/s12888-019-2175-7 (PMC6599349; doi:10.1186/s12888-019-2175-7)
Supplement: Supplementary file 1 — Glossary for HoNOS Score Sheet. (PDF 353 kb) [file 12888_2019_2175_MOESM1_ESM.pdf]

HoNOS

Health of the Nation Outcome Scales

RC PSYCH
ROYAL COLLEGE OF PSYCHIATRISTS

Glossary for HoNOS Score Sheet

Royal College of Psychiatrists
6th Floor, Standon House, 21 Mansell Street, London E1 8AA

HoNOS August 1996

Authors: J. K. Wing, R. H. Curtis, A. S. Beevor
Health of the Nation Outcome Scales
(HoNOS) © Royal College of Psychiatrists

Summary of rating instructions:

1) **Rate each scale in order from 1 to 12**
2) **Do not include information rated in an earlier item except for item 10 which is an overall rating**
3) **Rate the MOST SEVERE problem that occurred during the period rated**
4) **All scales follow the format:**
   - **0 = no problem**
   - **1 = minor problem requiring no action**
   - **2 = mild problem but definitely present**
   - **3 = moderately severe problem**
   - **4 = severe to very severe problem**

Rate 9 if not known

1. Overactive, aggressive, disruptive or agitated behaviour

- *Include such behaviour due to any cause, e.g. drugs, alcohol, dementia, psychosis, depression, etc.*
- *Do not include bizarre behaviour rated at Scale 6.*

0 No problem of this kind during the period rated.
1 Irritability, quarrels, restlessness etc. not requiring action.
2 Includes aggressive gestures, pushing or pestering others; threats or verbal aggression; lesser damage to property (e.g. broken cup, window); marked overactivity or agitation.
3 Physically aggressive to others or animals (short of rating 4); threatening manner; more serious overactivity or destruction of property.
4 At least one serious physical attack on others or on animals; destructive of property (e.g. fire-setting); serious intimidation or obscene behaviour.

Rate 9 if not known

2. Non-accidental self-injury

- *Do not include accidental self-injury (due e.g. to dementia or severe learning disability); the cognitive problem is rated at Scale 4 and the injury at Scale 5.*
- *Do not include illness or injury as a direct consequence of drug/alcohol use rated at Scale 3 (e.g. cirrhosis of the liver or injury resulting from drink driving are rated at Scale 5).*

0 No problem of this kind during the period rated.
1 Fleeting thoughts about ending it all but little risk during the period rated; no self-harm.
2 Mild risk during the period rated; includes non-hazardous self-harm (e.g. wrist-scratching).
3 Moderate to serious risk of deliberate self-harm during the period rated; preparatory acts (e.g. collecting tablets).
4 Serious suicidal attempt and/or serious deliberate self-injury during the period rated.

Rate 9 if not known

3. Problem-drinking or drug-taking

- *Do not include aggressive/destructive behaviour due to alcohol or drug use, rated at Scale 1.*
- *Do not include physical illness or disability due to alcohol or drug use, rated at Scale 5.*

0 No problem of this kind during the period rated.
1 Some over-indulgence but within social norm.
2 Loss of control of drinking or drug-taking, but not seriously addicted.
3 Marked craving or dependence on alcohol or drugs with frequent loss of control, risk taking under the influence.
4 Incapacitated by alcohol/drug problem.

Rate 9 if not known

4. Cognitive problems

- *Include problems of memory, orientation and understanding associated with any disorder: learning disability, dementia, schizophrenia, etc.*
- *Do not include temporary problems (e.g. hangovers) resulting from drug/alcohol use, rated at Scale 3.*

0 No problem of this kind during the period rated.
1 Minor problems with memory or understanding (e.g. forgets names occasionally).
2 Mild but definite problems (e.g. has lost the way in a familiar place or failed to recognise a familiar person); sometimes mixed up about simple decisions.
3 Marked disorientation in time, place or person; bewildered by everyday events; speech is sometimes incoherent; mental slowing.
4 Severe disorientation (e.g. unable to recognise relatives), at risk of accidents; speech incomprehensible; clouding or stupor.

Rate 9 if not known

5. Physical illness or disability problems

- *Include illness or disability from any cause that limits or prevents movement, or impairs sight or hearing, or otherwise interferes with personal functioning.*
- *Include side-effects from medication; effects of drug/alcohol use; physical disabilities resulting from accidents or self-harm associated with cognitive problems, drink-driving, etc.*
- *Do not include mental or behavioural problems rated at Scale 4.*

0 No physical health problem during the period rated.
1 Minor health problems during the period (e.g. cold, non-serious fall, etc.).
2 Physical health problem imposes mild restriction on mobility and activity.
3 Moderate degree of restriction on activity due to physical health problem.
4 Severe or complete incapacity due to physical health problem.

Rate 9 if not known

6. Problems associated with hallucinations and delusions

- *Include hallucinations and delusions irrespective of diagnosis.*
- *Include odd and bizarre behaviour associated with hallucinations or delusions.*
- *Do not include aggressive, destructive or overactive behaviours attributed to hallucinations or delusions, rated at Scale 1.*

0 No evidence of hallucinations or delusions during the period rated.
1 Somewhat odd or eccentric beliefs not in keeping with cultural norms.
2 Delusions or hallucinations (e.g. voices, visions) are present, but here is little distress to patient or manifestation in bizarre behaviours, i.e. clinically present but mild.
3 Marked preoccupation with delusions or hallucinations, causing much distress and/or manifested in obviously bizarre behaviour, i.e. moderately severe clinical problem.
4 Mental state and behaviour is seriously and adversely affected by delusions or hallucinations, with severe impact on patient

Rate 9 if not known

7. Problems with depressed mood

- *Do not include overactivity or agitation, rated at Scale 1.*
- *Do not include suicidal ideation or attempts, rated at Scale 2.*
- *Do not include delusions or hallucinations, rated at Scale 6.*

0 No problem associated with depressed mood during the period rated.
1 Gloomy; or minor changes in mood.
2 Mild but definite depression and distress (e.g. feelings of guilt; loss of self-esteem).
3 Depression with inappropriate self-blame, preoccupied with feelings of guilt.
4 Severe or very severe depression, with guilt or self-accusation.

Rate 9 if not known

8. Other mental and behavioural problems

- *Rate only the most severe clinical problem not considered at items 6 and 7 as follows.*
- *Specify the type of problem by entering the appropriate letter;*
  A phobic; B anxiety; C obsessive-compulsive;
  D mental strain/tension; E dissociative; F somatoform;
  G eating; H sleep; I sexual; J other, specify.

0 No evidence of any of these problems during period rated.
1 Minor non-clinical problems.
2 A problem is clinically present at a mild level (e.g. patient has a degree of control).
3 Occasional severe attack or distress, with loss of control (e.g. has to avoid anxiety provoking situations altogether, call in a neighbour to help, etc.) i.e. moderately severe level of problem.
4 Severe problem dominates most activities.

Rate 9 if not known

9. Problems with relationships

- *Rate the patient's most severe problem associated with active or passive withdrawal from social relationships, and/or non-supportive, destructive or self-damaging relationships.*

0 No significant problems during the period.
1 Minor non-clinical problems.
2 Definite problems in making or sustaining supportive relationships: patient complains and/or problems are evident to others.
3 Persisting major problems due to active or passive withdrawal from social relationships and/or to relationships that provide little or no comfort or support.
4 Severe and distressing social isolation due to inability to communicate socially and/or withdrawal from social relationships.

Rate 9 if not known

10. Problems with activities of daily living

- *Rate the overall level of functioning in activities of daily living (ADL): e.g. problems with basic activities or self-care such as eating, washing, dressing, toilet; also complex skills such as budgeting, organising where to live, occupation and recreation, mobility and use of transport, shopping, self-development, etc.).*
- *Include any lack of motivation for using self-help opportunities, since this contributes to a lower overall level of functioning.*
- *Do not include lack of opportunities for exercising intact abilities and skills, rated at Scales 11-12.*

0 No problems during period rated; good ability to function in all areas.
1 Minor problems only (e.g. untidy, disorganised).
2 Self-care adequate, but major lack of performance of one or more complex skills (see above).
3 Major problems in one or more area of self-care (eating, washing, dressing, toilet) as well as major inability to perform several complex skills.
4 Severe disability or incapacity in all or nearly all areas of self-care and complex skills.

Rate 9 if not known

11. Problems with living conditions

- *Rate the overall severity of problems with the quality of living conditions and daily domestic routine. Are the basic necessities met (heat, light, hygiene)? If so, is there help to cope with disabilities and a choice of opportunities to use skills and develop new ones?*
- *Do not rate the level of functional disability itself, rated at Scale 10.*

NB: Rate patient's usual accommodation. If in acute ward, rate the home accommodation. If information not available, rate 9.

0 Accommodation and living conditions are acceptable; helpful in keeping any disability rate at Scale 10 to the lowest level possible, and supportive of self-help.

1 Accommodation is reasonably acceptable although there are minor or transient problems (e.g. not ideal location, not preferred option, doesn't like the food, etc.).

2 Significant problem with one or more aspects of the accommodation and/or regime (e.g. restricted choice; staff or household have little understanding of how to limit disability, or how to help use or develop new or intact skills).

3 Distressing multiple problems with accommodation (e.g. some basic necessities absent); housing environment has minimal or no facilities to improve patient's independence.

4 Accommodation is unacceptable (e.g. lack of basic necessities; patient is at risk of eviction or 'roofless'; or living conditions are otherwise intolerable) making patient's problems worse.

12. Problems with occupation and activities

- *Rate the overall level of problems with quality of day-time environment. Is there help to cope with disabilities, and opportunities for maintaining or improving occupational and recreational skills and activities? Consider factors such as stigma, lack of qualified staff, access to supportive facilities, e.g. staffing and equipment of day centres, workshops, social clubs, etc.*
- *Do not rate the level of functional disability itself, rated at Scale 10.*

NB: Rate patient's usual situation. If in acute ward, rate activities during period before admission. If information not available, rate 9.

0 Patient's day-time environment is acceptable: helpful in keeping any disability rated at Scale 10 to the lowest level possible, and supportive of self-help.

1 Minor or temporary problems (e.g. late giro cheques; reasonable facilities available but not always at desired times, etc.).

2 Limited choice of activities, e.g. there is a lack of reasonable tolerance (e.g. unfairly refused entry to public library or baths etc.); or handicapped by lack of a permanent address; or insufficient carer or professional support; or helpful day setting available but for very limited hours.

3 Marked deficiency in skilled services available to help minimise level of disability; no opportunities to use intact skills or add new ones; unskilled care difficult to access.

4 Lack of any opportunity for daytime activities makes patient's problems worse.

HoNOS Score Sheet

Scale 0-4 ***Rate 9 if not known***

1	Overactive, aggressive, disruptive or agitated behaviour		
2	Non-accidental self-injury		
3	Problem-drinking or drug-taking		
4	Cognitive problems		
5	Physical illness or disability problems		
6	Problems with hallucinations & delusions		
7	Problems with depressed mood		
8	Other mental & behavioural problems (specify A, B, C, D, E, F, G, H, I or J)		
9	Problems with relationships		
10	Problems with activities of daily living		
11	Problems with living conditions		
12	Problems with occupation and activities		

Total Score (0-48)
